# Study of the Scattering Effect by SiO_2_ Nanoparticles, in a Luminescent Solar Concentrator Sensitized with Carbon Dots

**DOI:** 10.3390/nano13172480

**Published:** 2023-09-02

**Authors:** Mackenson Polché, Blancas Flores José Miguel, Carlos Alberto Guzmán González, Gabriel González Contreras, Victor Hugo Romero Arellano

**Affiliations:** 1 Departamento de Agua y Energía, CUTonalá, Universidad de Guadalajara, Av. Nuevo Periférico No. 555 Ejido San José Tateposco, Tonalá 45425, Mexico; mackenson.polche@alumnos.udg.mx; 2 Departamento de Ciencias Básicas y Aplicadas, CUTonalá, Universidad de Guadalajara Av. Nuevo Periférico No. 555 Ejido San José Tateposco, Tonalá 45425, Mexico; jose.blancas@academicos.udg.mx (B.F.J.M.); alberto.guzman@academicos.udg.mx (C.A.G.G.); 3 Cátedras CONACYT, Coordinación para la Innovación y la Aplicación de la Ciencia y la Tecnología, Universidad Autónoma de San Luis Potosí, San Luis Potosí 78000, Mexico; gabriel.gonzalez@uaslp.mx

**Keywords:** carbon quantum dots, light scattering, SiO_2_ nanoparticles, photoluminescence, luminescent solar concentrators

## Abstract

Luminescent solar concentrators (LSCs) have become an attractive way to produce green energy via their integration into buildings as photovoltaic windows. Recently, carbon quantum dots (C-QDs) have become the most studied luminescent material for the manufacture of luminescent solar concentrators due to their advantages, such as low toxicity, sustainability, and low cost. Despite the advantages of carbon quantum dots, they remain a low-efficiency material, and it is difficult to fabricate LSCs with a good performance. To address this problem, some of the research has used SiO_2_ nanoparticles (Nps) to produce a light-scattering effect that helps to improve the system performance. However, these studies are limited and have not been discussed in detail. In this regard, this research work was designed to evaluate the contribution of the scattering effect in different systems of carbon quantum dots used in a possible luminescent solar concentrator. To carry out this study, C-QDs and SiO_2_ Nps were synthesized by hydrothermal methods and the Stober method, respectively. We used different concentrations of both materials to fabricate film LSCs (10 × 10 cm^2^). The results show that the light scattered by the SiO_2_ Nps has a double contribution, in terms of light redirected towards the edges of the window and as a secondary source of excitation for the C-QDs; thus, an improvement in the performance of the LSC is achieved. The best improvement in photoluminescence is achieved when the films are composed of 20% wt carbon quantum dots and 10% wt SiO_2_ Nps, reaching a gain of 16% of the intensity of the light incident on the edges of the window with respect to the LSCs where only C-QDs were used.

## 1. Introduction

Luminescent solar concentrators (LSCs) have become an attractive technological area for solar energy conversion devices that can integrate with modern urban architecture [1,2,3]. Generally, a luminescent solar concentrator consists of a polymeric film doped with luminescent materials called chromophores [4]. These chromophore materials can absorb different wavelengths from incident solar radiation; they excite and then re-emit the downshifted light by luminescence [5]. Finally, the downshifted light is directed to the small edges of the LSCs, directly or by total internal reflection, where it is collected and converted into electricity by integrated solar cells [1]. The most used materials in LSCs are lanthanides, organic dyes, and quantum dots [6,7]. The latter are divided into the quantum dots derived from metals such as CdSe, PbS, and ZnSe and the organic quantum dots or carbon quantum dots. Metal-based quantum dots can be highly efficient luminescent materials; however, these materials have negative impacts on the environment and health [8,9]. Furthermore, with metallic quantum dots, after a certain time of use, the metallic ions become toxic materials for the environment and for health [10]. Recently, many researchers have been very interested in carbon quantum dots (C-QDs) because these luminescence materials have certain environmental, health, and sustainability advantages. These luminescent materials contain low toxic elements; they differ from metallic quantum dots [11,12,13]; furthermore, the synthesis methods, like solvothermal, microwave, and carbonization, are generally easy and relatively cheap [14]. Due to these properties, carbon quantum dots have been used to explore various applications, such as bio-imaging and biomedical applications [9,11], photocatalysis [11], light-emitting diodes (LEDs) [7], displays [11], photovoltaics [7], and chemical sensing [12], among others.

Despite their many advantages, the quantum emission yield and brightness of organic-based quantum dots suffer due to their high sensitivity to contaminants in their environment; this is due to the presence of carboxyl groups that form non-radiative recombination centers. To deal with this issue, surface passivations have had very favorable results; for this strategy, a wide variety of both organic and inorganic materials has been used; in addition, different polymers have been used that have the function of forming a thin insulating capping layer that shields C-QDs from the adhesion of impurities and further improves their fluorescence [15,16]. It has been reported that carbon dots immersed in polymeric films exhibited enhanced fluorescence emission due to the passivation attributed to the polymeric matrix [17,18]. This has provided the opportunity to consider C-QDs as potential materials to be implemented in LSCs, which are also attractive because they have a broad excitation band in the near-ultraviolet–visible (UV-Vis) region, with strong emissions in the visible region [13].

LSCs based on C-QDs have been reported in different configurations, including the single-layer configuration, which is the most basic; it involves incorporating the C-QDs and embedding them into a polymer matrix [19]. Another configuration that has become popular is tandem-structured LSCs. Unlike the single-layer configuration, this configuration uses two or more overlapping layers, each with different types of C-QDs, thereby expanding the absorption and emission response range of the system [20]. Similarly, laminated LSCs based on C-QDs are an adaptation of the single-layer configuration and include the adding of another glass slide placed on top of the C-QDs/polymeric film; with this configuration, the *G* factor is defined as the ratio of the area of the top surface and the surface of the edge that is favored; thus, it is possible to improve the optical efficiency [21]. In addition to these configurations, different strategies have been explored to improve the response of these systems based on C-QDs, such as the use of plasmonic metal nanoparticles to improve the optical absorption and emission of C-QDs or the use of hybrid systems with organic/metallic quantum dots, among others [22,23,24].

On the other hand, there are different loss mechanisms in LSCs; among the most common are front surface reflection, transparency to long-wavelength incident light, reabsorption, escape cone losses, and scattering loss [25]; the latter refers to the light scattered by the embedded particles in the system, redirecting photons into the escape cone. However, not all the scattered light becomes a loss; it may also represent collected light gains since part of the scattered light reaches the edge of the CSL directly or by total internal reflection. The light-scattering phenomenon from nanostructures has been used as a strategy to optimize different systems, such as a light-scattering layer used in photovoltaic solar cells, or to improve the optical performance of LEDs and luminescent materials [26,27,28].

In 2010, Chau et al. [29] defined the “transparent solar cell window module” as a device analogous to CSLs, with the difference that instead of luminescent materials, it incorporates TiO_2_ nanoparticles in a polymethyl methacrylate (PMMA) matrix into the system. With this configuration, a part of the solar radiation is transmitted, while another part is redirected towards the edge of the window due to the light scattering caused by the TiO_2_ Nps. Using the same idea, Feng Zhang et al. [30] report a study of “transparent scattering solar concentrator” based on aligned nanosheets of SiO_2_ aerogel.

In the case of solar concentrators, this strategy has been underexplored; Debije et al. [31] report a configuration that adds a white scattering layer to the bottom side of the LSCs in order to improve the absorption of the dye by allowing a second passage of unabsorbed light due to the scattered light from the bottom layer. Zhao et al. [32] used silica microspheres dispersed at the PVA film surfaces to pair the total internal reflection in flexible films with the photoluminescent carbon quantum dots embedded in them. Another way to take advantage of the light-scattering phenomena was reported by Liu et al. [33]; this method was replicated by other authors [34], who, in addition to the luminescent material, incorporated SiO_2_ or TiO_2_ nanoparticles as scattering material to increase the probability of the light captures and absorbance by QDs. Our system involves the use of a nanocomposite polymeric film with C-QDs as a fluorescent emitter and SiO_2_ nanoparticles as light-scattering material so that the carbon quantum dots receive double excitation. The first is by the direct incidence of solar radiation, and the second is due to the scattering effect produced by the silicon particles. In addition, the scattered light also contributes as an incident light at the edges of the LSCs. In this way, using SiO_2_ particles can enhance the LSC devices´ efficiency. In the previous research, they were interested in quantifying the efficiency of luminescent solar concentrators using light-scattering particles, such as TiO_2_ or SiO_2_ Nps [32,33]. However, these systems (LSCs) were focused on the characterization of electrical parameters such as the current density, the voltage, and the performance efficiency. None of these works describes which of the incident wavelengths are those at the edges of the window and which of those wavelengths were favored by the light-scattering phenomenon, as well as other optical phenomena such as the total internal reflection and luminescence of the C-QDs. Knowing the nature of optical phenomena and their contribution to the incident light on the edges of the LSCs will allow a better choice of materials in the manufacture of composite films where luminescent particles and light-scattering particles are combined as an improvement strategy in LSCs. This study was designed to evaluate the contribution of the light-scattering effect in different LSC systems. To develop this research, two nanomaterials were synthesized: C-QDs and SiO_2_ Nps. These nanomaterials were used in different concentrations to fabricate polyvinylpyrrolidone (PVP) nanocomposite films; deposited on a glass substrate, the different LSCs were placed vertically by means of a support, and a halogen lamp was an external light source placed in front of the LSCs. In order to evaluate the light-scattering effect of the SiO_2_ Nps, the different wavelengths incident at the edge of the window were collected using a collimating lens coupled to an optical fiber connected to a spectrofluorometer. Under this experimental arrangement, the contribution of the different optical phenomena of the system is discussed.

## 2. Materials and Methods

### 2.1. Materials

To carry out this research, the following materials were used: urea (CON_2_H_4_), citric acid (C_6_H_8_O_7_), dimethylformamide (DMF), hexane (C_6_H_14_), methanol (CH_3_OH), sodium hydroxide (NaOH), 2-propanol (C_3_H_8_O), deionized water (H_2_O), ammonium hydroxide (NH_4_OH), tetraethoxysilian (TEOS) (purity ≥ 99.0%), and polyvinylpyrrolidone (PVP). All the reagents were purchased from Sigma-Aldrich.

#### 2.1.1. Synthesis of C-QDs

The C-QDs were synthesized under the hydrothermal/solvothermal method, following previously reported methodologies [35,36]. A solution of 1 g of citric acid, 2 g of urea, and 10 mL of DMF was prepared in a beaker. The solution was placed on a magnetic plate with the objective of stirring until all the reagents were dissolved. Once the solution was found to be homogeneous, it was placed in an autoclave reactor. The autoclave was put in an oven to heat the solution to 160 °C for 6 h. At the end of the time, the dark orange solution (C-QDs) was precipitated by dropping in 25 mL of hexane. The precipitate was recovered and most of the hexane was removed. Then, the solution was dispensed again in 60 mL of methanol and shaken until a homogeneous solution was obtained. A second solution of 20 mL of 1.25 M NaOH was prepared, added to the first, and stirred for 1 minute. The new solution was precipitated dropwise in 25 mL of hexane, and the precipitate was recovered; then, the solution was dispersed in 60 mL of methanol to obtain homogenization of the sample. The sample was centrifuged at 6000 RPM for 10 min, and the supernatant was recovered and saved for posterior characterization and analysis.

#### 2.1.2. Synthesis of SiO_2_ Nps

The Stober method was used for the synthesis of the SiO_2_ Nps. For the preparation of the SiO_2_ Nps, a Schott beaker was used to make a solution of 32 mL 2-propanol, 12 mL deionized water, and 6.5 mL NH_4_OH, and the solution was vigorously stirred by an electric magnetic stirrer for 5 min. Afterwards, 300 µL of TEOS was added, and the mixture was capped and allowed to shake for 30 min. At the end of this time, a slight opalescent tone was observed. Then, 1.25 mL of TEOS was added dropwise and allowed to stir for 60 min. At the end of 60 min, a white liquid was obtained. Finally, to obtain the SiO_2_ Nps, the solution was centrifuged 2 times: the first time for 10 min at 6000 RPM and the second time for 5 min, dispersed in deionized water. Subsequently, to dry them, they were left in an oven at 60 °C for 24 h.

#### 2.1.3. Fabrication of Luminescent Solar Concentrator

Three different types of luminescent solar concentrators were fabricated: LSCs based on C-QDs, LSCs based on SiO_2_ Nps, and LSCs based on a mix of C-QDs and SiO_2_ Nps. Commercial glass (10 × 10 × 0.13 cm) was used. First, we prepared a solution of PVP (PVP K30 concentration: 200 mg mL) in 7 mL of methanol with moderate agitation. Then, different concentrations of C-QDs were dispersed in the PVP solution and sonicated for 10 min to homogenize. Finally, to obtain different LSCs based on C-QDs, the mixture was drop-casted onto the surface of a glass substrate. Before their characterization, the different samples obtained were dried at room temperature for 4 h in order to evaporate the solvent. For the LSCs based on SiO_2_ Nps and based on a mix of C-QDs and SiO_2_ Nps, different volumes of C-QDs and SiO_2_ Nps were dispersed in 7 mL of PVP solution. Then, the same steps explained above were followed. Thus, three different types of LSCs (LSC-CQD; LSC-SiO_2_; and LSC-CQD-SiO_2_) with different volume concentrations were obtained.

### 2.2. Characterization Techniques

**Figure 1:**
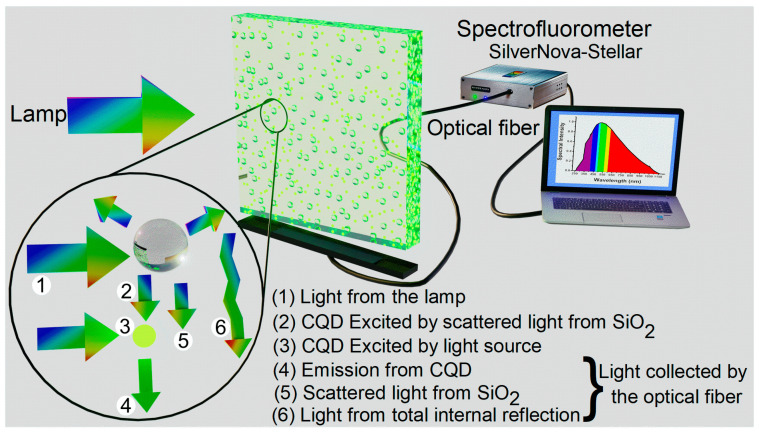
Schematic illustration of the luminescent solar concentrator with film composed of C-QDs and SiO_2_ Nps. The optical phenomena of the interaction of an external light source with the LSC are shown in the representative magnification circle. The contribution of incident light at the edges of the LSC is collected by optical fiber and analyzed by spectroscopy.

The morphologies of the C-QDs and SiO_2_ Nps were analyzed using a TESCAN model Mira 3 LMU scanning electron microscope. A Cary-700 UV–Vis-NIR spectrophotometer was used to obtain the characterization of the absorption and transmission spectra. Photoluminescence (PL) of the C-QDs was performed using a spectrofluorometer (FluoroMaxPlus-C, Horiba Scientific). The analysis of the optical phenomena of the different systems was carried out by placing the LSCs vertically using a homemade support placed on the lower edge of the substrate. In order to collect incident light at the edges of the LSCs, on the dais support, we adapted a collimating lens connected to the fiber optic cable of the spectrofluorometer (Silver Nova-Stellar), and as an external light source, a lamp was used (Philips, halogen PAR 3200Cd), placed in the front at a distance of 20 cm. All the experimentation was conducted in a dark room at room temperature. Figure 1 shows the design of the experiments. When the light from the excitation lamp falls on the luminescent solar concentrator, it interacts with the different materials and gives rise to different optical phenomena in the system. These optical phenomena are shown in the representative magnification circle. When light from the external source is absorbed by the C-QDs, photons are re-emitted by down-conversion, and the emitted light can reach the edges of the device directly or by total internal reflection. As for the SiO_2_ Nps, a large part of the light that falls on them is scattered in all directions; in the same way, this scattered light reaches the edge directly or by total internal reflection. However, the scattered light can be found in Q-CDs in the path; therefore, the Q-CDs receive double excitation, one from the external light and the other from the scattering effect produced by the SiO_2_ Nps. Finally, all these wavelengths that reach the edges of the device are collected by the optical fiber and sent to the computer for further analysis.

## 3. Results and Discussion

**Figure 2:**
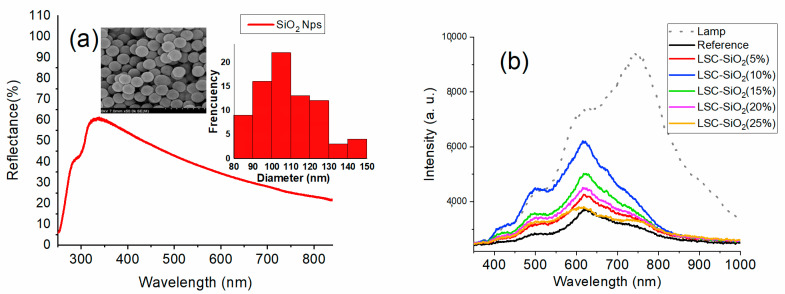
(**a**) Spectral reflectance of SiO_2_ Nps; inset shows SEM image and size distribution. (**b**) Photoluminescence spectra of LSC-SiO_2_ systems at different SiO_2_ concentrations.

The spherical SiO_2_ Nps synthesized by the Stöber method are shown in the SEM micrographs in the inset of Figure 2a. The average diameter of the silicon spheres was 100 nm. The spectral reflectance in Figure 2a shows a broadband that begins around 300 nm, with a maximum of 60% reflectance in the region centered at 350 nm; later, the curve begins to descend for longer wavelengths. However, it maintains reflectance values above 30%. This characteristic optical response of this type of particle has made SiO_2_ spheres good candidates as light-scattering agents in different systems [37,38].

As described in the experimental section, the optical fiber mounted in the LSC system is responsible for collecting the different light beams that are directed towards one of the edges of the LSC, for which the emission spectra obtained are the enveloping of the overlap of the different optical phenomena participating in the system. In order to break down the contribution of each of the elements of the composite films, Figure 2b first shows the spectra of light collected from the different LSC-SiO_2_. For the reference sample (PVP film without Nps), the spectrum of light in the region from 380 nm to 700 nm exhibits almost the same structure as the radiation spectrum of the external light source (dotted curve); therefore, it is assumed that a part of the light from the incident source travels towards the edge of the window due to the phenomenon of total internal reflection. Subsequently, due to the light-scattering phenomenon, the intensity of the curve increases as the volume of the SiO_2_ Nps in the LSC film increases, until reaching a maximum of 10% SiO_2_ Nps. Subsequently, the intensity of the curve begins to decrease due to the saturation of the Nps, which propitiate an obstruction of the free passage of the scattered light towards the edge of the LSC. It can be seen that the main peak of radiation from the lamp, centered at 800 nm, decreased considerably, which indicates that this region is mostly transmitted through the LSC, which is consistent with the reflectance spectrum discussed in Figure 2a.

**Figure 3:**
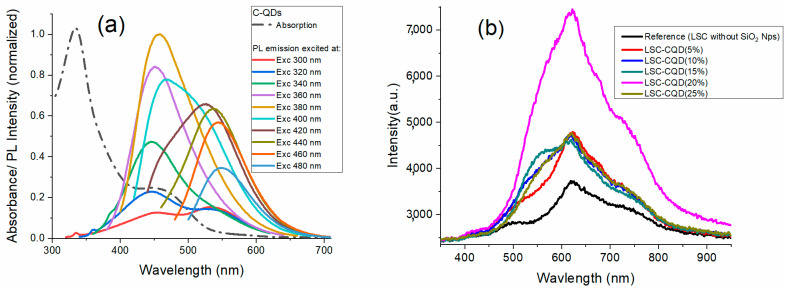
(**a**) Absorption and emission spectra of C-QDs under different excitation wavelengths (**b**) photoluminescence spectra of LSC-CQD systems at different C-QDs concentrations.

The absorption and emission spectra of the C-QDs are shown in Figure 3a. The absorption curve (dotted curve) shows a main peak in the UV region, centered at 334 nm, as well as a secondary band centered at 470 nm that extends to 550 nm. These are attributed to the intrinsic absorption of the *n* → π* transition of the C=O bond in the carbon cores and the transition from the surface state with lone electron pairs, respectively [39,40]. The emission spectra exhibit two principal peaks under different excitation wavelengths, one centered at 450 nm and the other centered at 560 nm. It can be seen that in the region from 420 nm to 550 nm the absorption and emission spectra of the carbon quantum dots overlap. This overlap translates into a loss of emission intensity due to the reabsorption phenomenon, which is evidenced in Figure 3b, where the emission spectrum of the LSC-CQD systems is shown and in which it can be seen that in the said region there is no considerable contribution of PL due to the reabsorption. However, the C-QD emission band, in the region from 500 nm to 600 nm, is noticeable with respect to the reference LSC (the PVP film without C-QDs). It can be seen that the intensity of this band increases as the C-QD concentration increases, reaching a maximum for the LSC-CQD (20%) sample, and then decays due to Q-CD saturation. Subsequently, above 600 nm, nonlinear behavior occurs, which is possibly due to the combination of phenomena among the different luminescence intensities of the C-QDs, the supersaturation of the Q-CD concentration, and the change in the refractive index of the film due to the concentration of Q-CDs [41,42,43].

**Figure 4:**
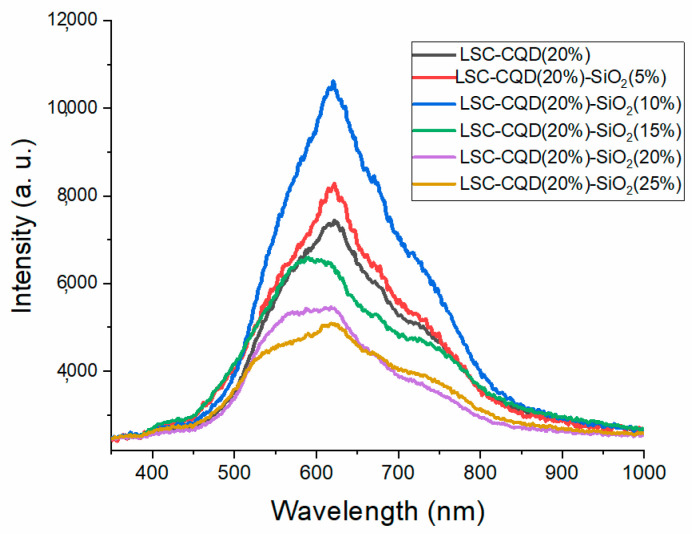
Photoluminescence spectra of LSC-CQD-SiO_2_ systems.

To analyze the contribution of the light-scattering phenomenon in the LSC-CQD-SiO_2_ systems, the C-QD concentration with the best performance (LSC-CQD 20%) was chosen in combination with different concentrations of SiO_2_ Nps. The emission spectra of these systems are shown in Figure 4. For sample LSC-CQD(20%)-SiO_2_(5%), the intensity has a slight increase with respect to the sample without SiO_2_ Nps; later, for LSC-CQD(20%)-SiO_2_(10%), the maximum intensity is achieved. However, as the SiO_2_ amount increases, the system is affected, and the intensities decrease. This behavior is attributed to the saturation of the system, where the same particles represent a blockage that decreases the amount of light that reaches the edges of the glass; however, it can be observed that the quenching of the curves occurs at a different rate between the bands’ centers at 550 nm and 750 nm, which is due to the fact that the 550 nm band corresponds to the emission of the Q-CDs that receive double excitation, from the incident light from the external source and from the light scattered by the SiO_2_ particles.

**Figure 5:**
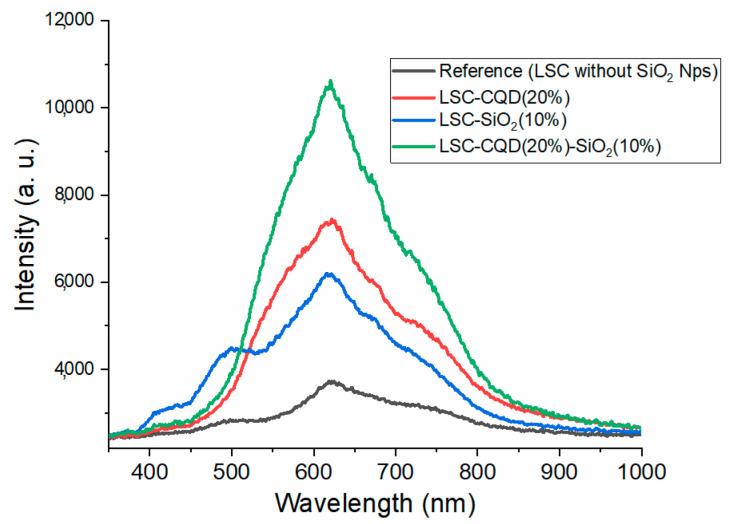
Comparative photoluminescence spectra of different LSCs systems.

In order to obtain a better comparison, Figure 5 presents the curves with the highest intensity of each of the LSCs systems; it can be seen that the curve with the highest intensity corresponds to the LSC-CQD(20%)-SiO_2_(10%), reaching an increase of 16% of the total area under the curve with respect to the LSC-CQD(20%) sample. In the same way, it can be observed that the curve centered at 500 nm on the LSC-SiO_2_ spectra is not present in the PL curves of the LSC-CQD and LSC-CQD-SiO_2_ samples: this corroborates the fact that this band has been absorbed by the C-QDs as a second source of excitation, thus causing an increase in the intensity of the sample LSC-CQD(20%)-SiO_2_(10%).

According to what has just been discussed, it is important to note that the spectrum of light scattered by the silicon particles must coincide with the C-QDs’ excitation region; in this way, the versatility of what is presented in this work is remarkable since, according to the Mie theory in corroboration with different works [44,45], the region of scattered wavelengths can be tuned as a function of the size and shape of the scattering material, thus adapting to the needs of the fluorophore or quantum dots under study. In this sense, an extension of this work could be carried out using different sizes of SiO_2_ particles.

**Figure 6:**
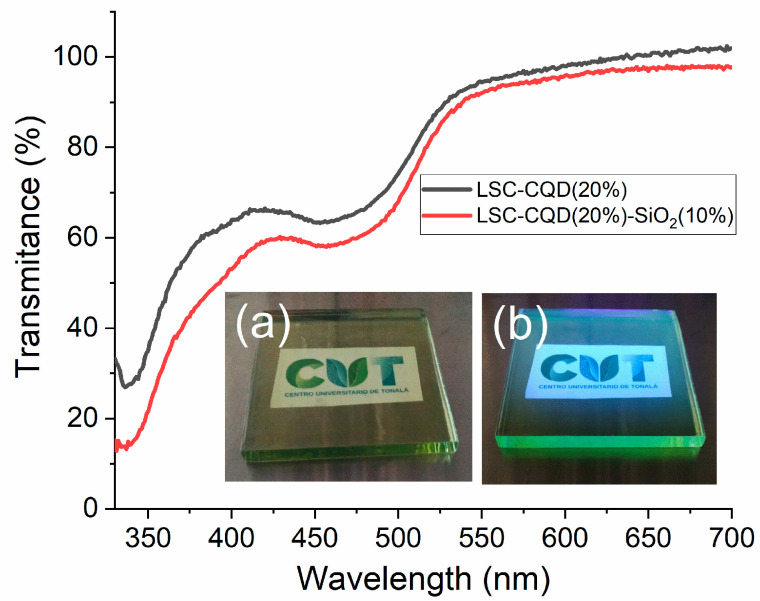
Comparative transmittance spectra of LSC-CQD(20% and LSC-CQD(20%)-SiO_2_(10%). The inset shows the transparency of the LSC (**a**) under ambient light and (**b**) under UV light.

On the other hand, the transmittance spectra of the LSC-CQD and LSC-CQD-SiO_2_ systems are shown in Figure 6. It can be seen that for the LSC-CQD the transmittance is above 60% in the entire visible region, and by incorporating the SiO_2_ particles into the system (LSC-CQD-SiO_2_,) the transmittance decreases only 3% with respect to the LSC-CQD. The inset shows the qualitative transparency of the sample, which makes it viable for use in buildings. However, it is known that one of the greatest difficulties of the LSCs is the scaling of the system since the photons that contribute to the efficiency of the CSSL at greater travel distances increase the probability of falling into the escape cone; recently, however, in addition to the application in buildings, other types of applications have been proposed for LSCs, where large-scale configurations are not required, such as photochemical or electrochemical reactors and microalgal production [2,46,47]. With this analysis, it can be deduced that the incorporation of light-scattering particles in LSC systems is a strategy with a high potential to increase the performance of these devices; therefore, the present study can be extended to other types of particles or systems where the phenomenon of light scattering is exploited.

## 4. Conclusions

In summary, in this study we analyzed the enhancement of the photoluminescence in LSCs sensitized by C-QDs through the scattering effect produced by SiO_2_ Nps. The experimental arrangement allowed the breaking down of the incident radiation at the edge of the LSC; this was attributed to different optical phenomena, including the light scattering due to the SiO_2_ Nps, the total internal reflection, and the broadening of the C-QD emission, due to a double excitation by the external source and by the light scattered from the SiO_2_ Nps.

Understanding the contribution of these phenomena will allow a better exploration of these systems with other types of light-scattering particles. The results of this work showed that the LSC-CQD(20%)-SiO_2_(10%) systems reached a 16% increase in the intensity of incident light at the edge of the LSC with respect to the sample where only C-QDs were used. Therefore, the use of the phenomenon of scattering is a strategy with great potential in LSC applications. In order to achieve a better understanding of this study, different characterizations, such as the scattering loss, the quantum efficiency, the scaling of the LSCs´ sizes, and their long-term stability, among others, should be analyzed in future work.

## Data Availability

The data presented in this study are available on request from the corresponding author.
